# The Relationship Among Trait Mindfulness, Attention, and Working Memory in Junior School Students Under Different Stressful Situations

**DOI:** 10.3389/fpsyg.2021.558690

**Published:** 2021-03-02

**Authors:** Yuntao Li, Ningxi Yang, Yan Zhang, Wei Xu, Li Cai

**Affiliations:** ^1^Psychological Education and Counselling Centre, Southwest University of Science and Technology, Mianyang, China; ^2^Department of Applied Psychology, College of Humanities and Social Sciences, Harbin Engineering University, Harbin, China; ^3^Faculty of Psychology, Beijing Normal University, Beijing, China

**Keywords:** stress, trait mindfulness, attention, working memory, junior school student

## Abstract

Attention and working memory are important cognitive functions that affect junior school students’ learning ability and academic performance. This study aimed to explore the relationships among trait mindfulness, attention, and working memory and to explore differences in performance between a high trait mindfulness group and a low one in attention and working memory under different stressful situations. In study 1, 216 junior school students completed the Five Facet Mindfulness Questionnaire (FFMQ), and their attention and working memory were tested in a non-pressure situation. The results showed that attention had a partial mediating effect between mindfulness and working memory. In study 2, the high trait mindfulness group and the low one were tested for attention and working memory under situations with single and multiple pressures. One notable result was that the attention and working memory performances of the high mindfulness group were all significantly higher than those of the low mindfulness group in every stress situation (no stress, single stress, and multiple stresses). Other important results were that trait mindfulness moderates the relationship between stress and attention and between stress and working memory. These results suggest that trait mindfulness has a protective effect in the process by which various stresses affect attention and working memory. These findings indicate that trait mindfulness is an important psychological quality that affects the attention and working memory of junior school students, and it is also an important psychological resource for effectively coping with the impact of stress on attention and working memory. Therefore, it is possible that improving trait mindfulness may help to improve junior school students’ attention and working memory and enable them to cope better with stress, thereby helping to improve academic performance. This research is of great significance for understanding the association between key psychological qualities and cognitive functions in different stressful situations. These findings also provide insight for future studies in educational psychology.

## Introduction

Attention and working memory are two important and closely related cognitive functions. Attention refers to the ability to focus one’s psychological activities on something. Working memory is a system that temporarily stores and processes current information with limited capacity. As a cognitive resource, attention helps to deal with various tasks in the working memory system (Soto et al., 2005; Kumar et al., 2009; Dunning and Holmes, 2014). Previous studies have shown that both attention and working memory are closely related to students’ learning ability and performance (Durbrow et al., 2000; Ayres and Sweller, 2014). Issues surrounding students’ attention and working memory are currently attracting more and more attention as topics of research (Rebok et al., 2014; Rode et al., 2017; Wimmer et al., 2020). The question of which psychological or external factors affect attention and working memory has also become a topic of discussion.

In recent years, mindfulness has attracted more and more attention in psychological research (Davidson, 2010; Brown et al., 2015; Lindsay and Creswell, 2017). Mindfulness can be viewed as both a state and as a special characteristic. State mindfulness describes the non-judgmental, present-focused awareness experienced in any given moment (Medvedev et al., 2017). Trait mindfulness or dispositional mindfulness involves stable individual differences in average frequency and intensity of mindful states over time (Brown and Ryan, 2013; Jamieson and Tuckey, 2017; Mesmer-Magnus et al., 2017). It has been shown that trait mindfulness can be improved by practice, which includes mindfulness meditation as well as some mindfulness-based interventions (Cavanagh et al., 2014; Kiken et al., 2015; Quaglia et al., 2016). Mindfulness practice also has positive effects on attention and working memory. Some studies have found that mindfulness training can improve children’s sustained attention (Tarrasch, 2018), protect working memory capacity (Bailey et al., 2020), and significantly improve working memory performance (Chambers et al., 2008). It can thus be inferred that high trait mindfulness may also have positive impacts on attention and working memory.

People face pressures from different aspects of modern society. According to the Yerkes–Dodson law, moderate pressure will make individuals concentrate more energy to achieve better learning and to work efficiently (Yerkes and Dodson, 1908). However, when an individual is faced with stressful situations beyond their psychological capacity, it is difficult to make effective adjustment, resulting in negative emotions such as anxiety and depression. These in turn have adverse effects on cognitive functions such as attention and hinder the completion of tasks (Myhr et al., 2019). Working memory, as a cognitive resource with limited capacity, is more vulnerable to such effects (Matthews and Campbell, 2010; Lukasik et al., 2019).

In recent years, some studies have found that trait mindfulness is a positive psychological trait that can reduce stress sensitivity (Allen et al., 2015; Lomas et al., 2017) and the impairment of cognitive function by stress. Mindfulness meditation and high levels of mindfulness can significantly reduce the damage done to the memory of college students under stressful situations and can enable the subjects to complete the corresponding tasks more stably (Limaree, 2007). Another study was conducted on soldiers who were going to be sent to the battlefield in Iraq. The results showed that among these soldiers facing great pressure, those without psychological intervention showed a decrease in working memory capacity as the departure time approached. However, among the soldiers who received an 8-week mindfulness training, the working memory capacity of those who spent more time in self-training after class did not decrease significantly. This suggests that a certain intensity of mindfulness practice can to an extent protect working memory in the face of high-stress situations (Jha et al., 2010, 2015).

In China, junior school students are generally faced with heavy academic tasks and the pressure of gaining entry to high school. What are the effects of different stress levels on the attention and working memory of junior school students? Does trait mindfulness, as a positive psychological factor, regulate the effect of stress on junior school students’ attention and working memory? These problems are of great practical significance for junior school students and other groups facing high pressure. However, so far, studies on the moderating effect of trait mindfulness on attention and working memory in stressful situations and the relationships among trait mindfulness, attention, and working memory have still been extremely rare, especially in the context of junior school students, for which no relevant research reports have been found. Therefore, this research was divided into two parts to explore these issues. In study 1, the relationship among trait mindfulness, attention, and the working memory of junior school students and the internal mechanism of that relationship were explored. In study 2, stressful situations were created in an attempt to explore whether trait mindfulness has consistently positive effects on attention and working memory across a range of stressful situations, and in particular, whether trait mindfulness has moderating effects on attention and working memory in stressful situations.

The following three hypotheses were thus proposed:

H1: Trait mindfulness can predict working memory through the mediating effect of attention.

H2: In a given stress situation (no stress, single stress, or multiple stresses), there is a significant difference in the junior school students’ attention and working memory performance between the high trait mindfulness group and the low one.

H3: Trait mindfulness has a moderating effect on the influence of different intensities of stress on attention and working memory.

This research enriched the theoretical research on the influencing factors of attention and working memory. From the perspective of key psychological traits, it provides important clues toward, and a scientific basis for, improving the level of attention and working memory of individuals in stressful situations and thereby improving academic performance, and it has practical significance for carrying out effective education in schools.

## Study 1

This research was approved by the Harbin Engineering University Ethics Committee. All subjects were informed before the test of the purpose and method of the research and their right to participate. It was explained that participation was voluntary and anonymous and that there would be no penalty for withdrawing from the study at any stage without notice. The written informed consent of the subjects was obtained for all studies.

In study 1, a cross-sectional study was conducted to investigate the correlation among mindfulness, attention, and working memory in junior school students and to explore the mediating role of attention in the influence of mindfulness on working memory.

### Methods

#### Participants and Procedures

Two hundred and seventeen junior school students who voluntarily participated in the study were studied by the convenience sampling method, and 216 valid data were finally obtained. The age range of the subjects was 9–15 years old (*M* = 11.88, SD = 1.20). The distribution of gender and grades was as follows: 131 boys (60.65%), 85 girls (39.35%); 106 in grade 1 (49.07%), 71 in grade 2 (32.87%), and 39 in grade 3 (18.06%). All subjects had normal intelligence and normal or corrected vision.

All studies were conducted in a quiet environment without interference or stress. The examiner used uniform instructional language to explain the purpose and specific requirements of the test. After ensuring that the subject understood the instructions, they were tested in groups for the mindfulness trait. Then individual tests of attention were performed, and 3 days later, individual tests of working memory were performed to avoid fatigue effects.

#### Measures

##### Trait mindfulness test

The Five Facet Mindfulness Questionnaire (FFMQ) is an established questionnaire that is used to assess trait mindfulness (Baer et al., 2006). In the current study, the Chinese version of the FFMQ, as revised by Deng et al. (2011), was used. It consists of 39 items rated on a five-point Likert scale from 1 = *never or very rarely true* to 5 = *very often or always true*. The FFMQ is composed of five subscales, including “observe,” “describe,” “actaware,” “non-judge,” and “non-react.” Item scores are summed to form a mindfulness score, with higher scores indicating higher levels of trait mindfulness. In this study, the Cronbach’s alpha value for this scale was 0.744.

##### Attention test

**FIGURE 1:**
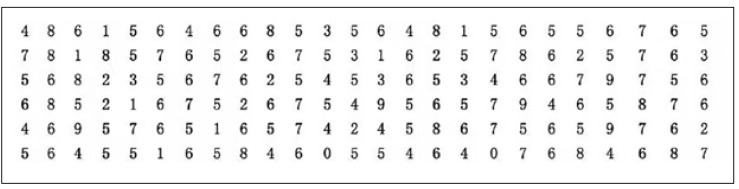
Schematic diagram of the digital cancelation test.

A traditional cancelation test is often used to test for attention ability (Schweizer et al., 2005). In this study, a paper-and-pen, designated digital cancelation test (Figure 1) was used to test the directivity and concentration of attention. The study material consisted of 1,000 Arabic digits (zero to nine). Subjects were asked to cross out the designated number 6 as quickly and accurately as possible. The ratio of the number of instances of the digit to be crossed out to the number of non-delimited digits was 1:4. The sorting distribution was uniform but not regular. The cancelation time (*T*) was recorded, and the cancelation accuracy was calculated as *A* = (*c* − *w*) / (*c* + *o*), where *c* represents the number of digits which were crossed out, *o* represents the number of digits which were missed, and *w* represents the number of digits which were crossed out incorrectly. The attention concentration index (*E* = *e* × *A*/*T*, where *e* represents the number of digits actually checked) was used as the final score indicator to measure attention. In this study, all the subjects completed the examination of 1,000 numbers, and thus, *e* = 1,000.

##### Working memory test

**FIGURE 2:**
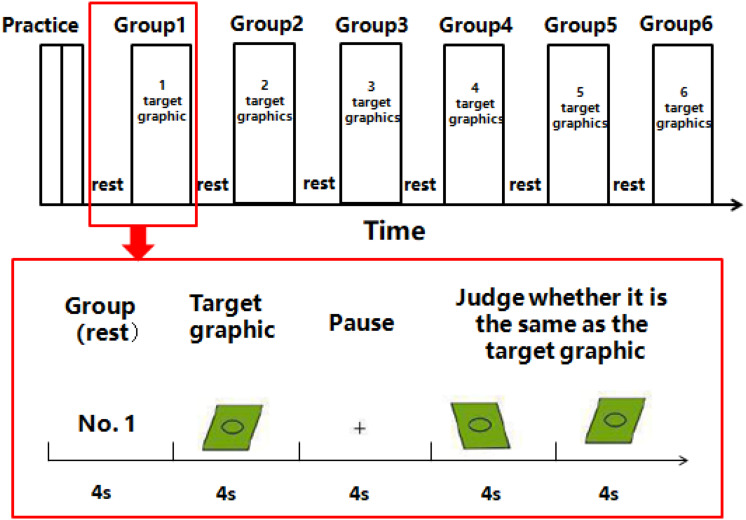
Schematic diagram of working memory test method (take the first group of tests as an example).

The level of visual working memory was evaluated by a classical, visual graphic working memory paradigm (Figure 2). The test task followed the definition of working memory given by Baddeley et al. (1996), that is, information needs to be processed and stored at the same time. The test was carried out on a computer with a screen size of 33.6 cm × 27 cm and a resolution of 1,280 × 1,024. The method was to present six groups of geometric graphics successively. From the first group to the sixth group, the number of target graphics gradually increased from one to six, and the graphics gradually changed from simple to complex. Each of the graphics was presented for 4 s, and the subjects were asked to observe and remember them quickly. When a “+” appeared on the screen, it meant that the presentation of the group of graphics was completed, and there would be a pause for 4 s. Next, the subjects were asked to judge whether the direction, shape, and size of each figure in the subsequent figure group were exactly the same as in the target figure just presented. Each correct answer counted for one point, and the total score was used as the visual working memory score.

#### Statistical Analysis

Descriptive statistical analysis was conducted on the mean value and standard deviation of attention, working memory, and the total score and facet scores of trait mindfulness for all subjects, and Pearson correlation coefficients were calculated to analyze the correlation between variables. In addition, based on controlling the influence of age on attention and working memory, the mediating effect model of attention on mindfulness and working memory was established by using the path analysis method. Descriptive statistics and correlation analysis were performed using SPSS 22.0 (IBM Corp., Armonk, NY, United States). AMOS 21.0 (IBM Corp., Armonk, NY, United States) was used for mediating effect analysis. Significance was established at *p* < 0.05.

### Results

#### Descriptive Statistics and Correlation Analysis of Each Variable

The average value, standard deviation, and correlation coefficient of trait mindfulness (including total score and five facet score), attention, and working memory are shown in Table 1. There was a significant positive correlation between total mindfulness score, attention, and working memory. The two mindfulness facets of “observe” and “actaware” were positively correlated with attention performance, and the three mindfulness facets of “observe,” “describe,” and “actaware” were positively correlated with work memory performance.

**TABLE 1:** Descriptive statistics and correlation analysis of mindfulness level, attention, and working memory of junior school students (*r*).

	*M*	SD	1	2	3	4	5	6	7	8
1. Observe	24.22	6.22	1							
2. Describe	22.20	4.36	0.15*	1						
3. Actaware	27.00	6.09	0.15*	0.23**	1					
4. Non-judge	22.57	4.82	0.37***	0.14*	0.10	1				
5. Non-react	19.52	4.04	0.47***	0.04	0.06	0.37***	1			
6. Total score of mindfulness	115.52	14.97	0.76***	0.50***	0.49***	0.57***	0.57***	1		
7. Attention	3.34	1.07	0.42***	0.10	0.52***	0.08	0.11	0.48***	1	
8. Working memory	36.64	11.86	0.41***	0.21**	0.51***	0.05	0.08	0.48***	0.55***	1

*p < 0.05, **p < 0.01, ***p < 0.001.

#### Analysis of the Mediating Effect of Attention

Path analysis was used to explore the mediating effect of attention on the relationship between mindfulness and working memory, by controlling the effect of age on attention and working memory. Because the “non-judge” and “non-react” facets had no significant correlation with attention and working memory, only the sum of the other three mindfulness facets was taken as a mindfulness score when constructing the mediating effect model.

**FIGURE 3:**
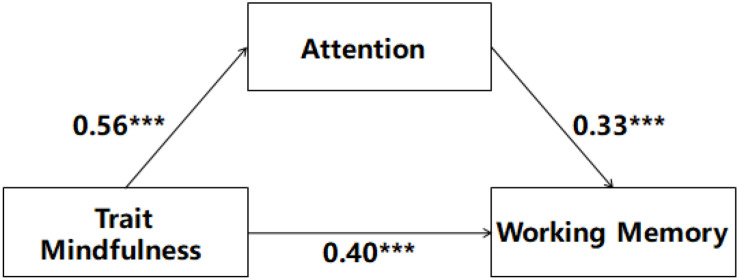
The relationship model of trait mindfulness, attention, and working memory. ***p < 0.001.

The results of the mediating effect model are shown in Figure 3. As the influence of age on attention and working memory was not significant (*p* < 0.05), the age is not indicated in Figure 3. The fitting index of this model was as follows: *χ*^2^/*df* = 2.976, GFI = 0.993, AGFI = 0.932, IFI = 0.990, TLI = 0.939, NFI = 0.985, CFI = 0.990. Therefore, the goodness of fit of this model was good. The non-parametric percentile bootstrap method of deviation correction was used to test the intermediate effect and estimate the confidence interval. The number of bootstrap repeated samplings was set to 5,000 and the confidence interval was set to 95%. The results showed that the confidence intervals of the mediating effects of this model do not contain zero, and the mediating effects were statistically significant (Table 2).

**TABLE 2:** Action path, effect size, and proportion of each variable in the model to working memory.

Path	Standardized effect value	Effect ratio	95% CI
Direct effect: mindfulness → working memory	0.40	68.40%	[0.28, 0.52]
Indirect: mindfulness → attention → working memory	0.56 × 0.33 = 0.18	31.60%	[0.12, 0.26]
Total effect	0.58		[0.49, 0.66]

Table 2 shows the action path, effect value, and proportion of all independent variables to dependent variables in this model. Among these, the effect value for the direct effect of mindfulness on working memory was 0.40, accounting for 68.40% of the total effect. The mediating effect of attention between mindfulness and working memory was 0.18, accounting for 31.60% of the total effect.

## Study 2

In this study, different stress situations were established to explore the effect of the interaction between stress and mindfulness on attention and working memory as well as further simple effects. A 2 (mindfulness level: low, high) × 3 (stress situation: none, single stress, multiple stresses) mixed experimental design was used. Among these, the level of mindfulness was the intersubject variable, and the stress situation was the intrasubject variable.

### Methods

#### Participants and Procedures

According to the total score of the FFMQ scale in study 1, participants were divided into high and low mindfulness groups. Among the test subjects, the highest scoring 27% was in the high mindfulness group, and the lowest scoring 27% was in the low mindfulness group. One hundred and sixteen junior school students participated in all the tests of study 2.

The test situation of study 1 was regarded as the stress-free situation, and the test results of study 1 were thus used as the attention and working memory performance scores of the subjects in the stress-free situation. Furthermore, each subject’s attention and working memory under single and multiple stresses were measured individually. All tests were conducted in a quiet environment. The examiner uniformly adopted the new instructional language to explain the purpose and specific requirements of the test. After ensuring that the subjects understood, the attention test was performed first and then the working memory test. To avoid the fatigue effect, subjects were given a 3-day interval between the attention test and the working memory test in the same stressful environment. In order to avoid the memory effect, for the same kind of test across different stress situations, subjects took parallel-form tests. In order to avoid the practice effect, the interval between the two similar tests was about 1 month.

#### Measures

##### Single stress situation

Time pressure was applied to the subjects in the single stress situation, in order to make individuals feel that it was challenging to complete the experimental task within a short time. The attention test required the subjects to complete the test within 1 min, and each picture in the working memory test was only presented for 2 s. When the examiner explained the instructions, they emphasized that the time constraints of the tests were significantly shorter than that of study 1, thereby giving the participants a sense of stress.

A copy of the number cancelation test was used as the experimental material to test attention in this situation. Compared with study 1, the completion time for the single stress situation was shortened to 1 min, the designated erasure number was adjusted from six to five, and all the number sorting was readjusted; other settings were unchanged.

A copy of the visual graphic working memory test was used as the experimental material to test working memory in this situation. Compared with study 1, the rendering time of the replica was shortened to 2 s, and the size, direction, or order of the target and alternative graphics were adjusted; other settings were unchanged.

##### Multiple stress situation

The premise of the multiple stress situation was to add situational pressure on top of time pressure, so that the subjects would feel the combined effect of more types of pressure. The time pressure was established in the same manner as described above. In China, learning ability and whether they are valued by teachers are important factors that affect the self-evaluation of junior school students. Therefore, when the examiner explained the instructions, it was emphasized that the participant’s teacher would be informed of the results of the attention and working memory test as an indicator to evaluate their learning ability, and the teacher would focus on training the top students in the follow-up teaching. At the end of all the experiments, the students were informed of the real situation, i.e., the test results were actually confidential and would not be disclosed to the teachers, nor would they influence the teachers’ evaluation and subsequent training of the students, so as to avoid any negative impact of the test on the students’ psychological state.

The interval between this test and the single stress situation test was 1 month, while the interval between this test and the study 1 test was 2 months. In order to avoid a memory effect of the test materials in the single stress situation, the attention and working memory experimental materials from study 1 were reused, but the time setting was the same as that in the single stress situation.

#### Statistical Analysis

Descriptive analysis of attention and working memory scores under different stress situations and at different levels of mindfulness was performed, with data expressed as mean ± standard deviation. The 2 × 3 mixed variance analysis was used to analyze the interaction effect and simple effect of the data. The independent variables were trait mindfulness and stress situation, and the outcome variables were attention and working memory. SPSS 22.0 (IBM Corp., Armonk, NY, United States) was used for analysis. The significance threshold was set at the *p* < 0.05 level.

### Results

#### The Influence of Stress Situation and Mindfulness Level on Achievement at the Attention Task

**FIGURE 4:**
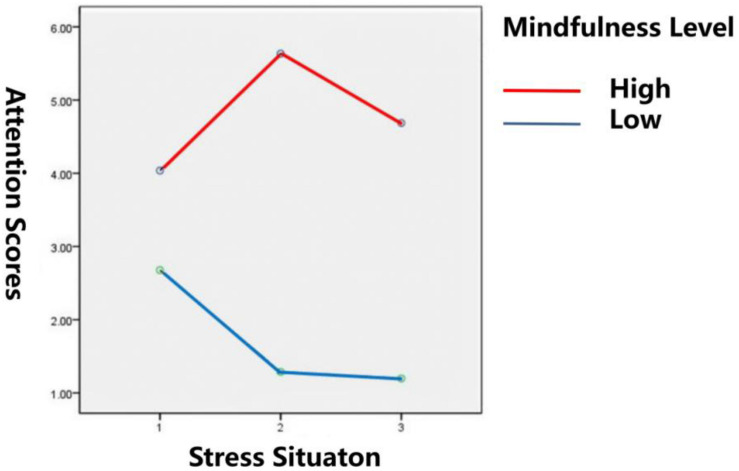
Interaction between stress situations and mindfulness levels on attention scores. 1 is stress-free situation, 2 is single stress situation, and 3 is a multiple stress situation.

The attention scores for different stress situations and mindfulness levels are shown in Table 3. The results of ANOVA showed that the interaction between mindfulness level and stress situation was significant [*F*(2,114) = 207.94, *p* < 0.001, η^2^_*p*_ = 0.646]. These results are presented in Figure 4. Further simple effect analysis showed that (1) there was a significant difference in the attention performance of the high mindfulness group under different stress situations [*F*(2,56) = 81.81, *p* < 0.001]. Further results showed that the attention performance of the high mindfulness group was significantly better in the single stress situation than in the multiple stress situation, and the attention performance in the multiple stress situation was also significantly higher than that in the stress-free situation. The attention level of the low mindfulness group decreased with the increase in stress, but the difference was not significant [*F*(2,56) = 26.51, *p* > 0.05]. (2) The attention scores of the high mindfulness group and the low one were significantly different in each stress situation. In a stress-free situation, *F*(2,56) = 54.61, *p* < 0.001. In the single stress situation, *F*(2,56) = 434.05, *p* < 0.001. Under the multiple stress situation, *F*(2,56) = 373.06, *p* < 0.001. These results show that in all three different stress situations, the attention performance of the high mindfulness group was significantly higher than that of the low mindfulness group.

**TABLE 3:** Attention scores under different stress situations and mindfulness levels (*M* ± SD).

		Stress situation		
		Stress-free situation	Single stress situation	Multiple stress situation
Mindfulness level	High mindfulness group (*n* = 58)	4.04 ± 1.22	5.64 ± 1.39	4.69 ± 1.15
	Low mindfulness group (*n* = 58)	2.68 ± 0.69	1.29 ± 0.78	1.20 ± 0.76

#### Effect of Stress Situation and Mindfulness Level on Working Memory Performance

**FIGURE 5:**
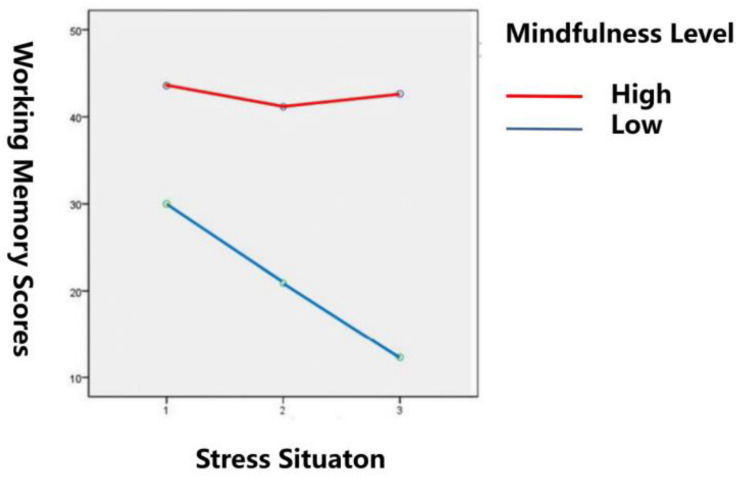
Interaction between stress situations and mindfulness levels on working memory scores.

The results of working memory under different stress situations and different mindfulness levels are shown in Table 4. The results of ANOVA showed that the interaction between mindfulness level and stress situation is significant (Figure 5), and *F*(2,114) = 72.31, *p <* 0.001, η^2^_*p*_ = 0.388. Further simple effect analysis showed that the working memory performance of the low mindfulness group was significantly different under different stress situations [*F*(2,56) = 258.28, *p <* 0.001]. Further results showed that the working memory performance of the low mindfulness group in the stress-free situation was significantly better than that in the single stress situation, and their working memory performance in the single stress situation was significantly higher than that in the multiple stress situation. There was no significant difference in the working memory scores of the high mindfulness group under different stress situations [*F*(2,56) = 2.21, *p* > 0.05]. The difference in working memory performance between the high mindfulness group and the low one in each stress situation was significant. In the stress-free situation, *F*(2,56) = 247.1, *p* < 0.001. In the single stress situation, *F*(2,56) = 247.1, *p* < 0.001. Under the multiple stress situation, *F*(2,56) = 550.21, *p* < 0.001. This shows that the working memory performance of the high mindfulness group was significantly higher than that of the low mindfulness group in all three different stress situations.

**TABLE 4:** Working memory performance under different stress situations and mindfulness levels (*M* ± SD).

		Stress situation		
		Stress-free situation	Single stress situation	Multiple stress situation
Mindfulness level	High mindfulness group (*n* = 58)	43.59 ± 1.47	41.16 ± 0.75	42.64 ± 1.01
	Low mindfulness group (*n* = 58)	30.00 ± 1.05	20.90 ± 1.05	12.33 ± 0.81

## Discussion

The results of study 1 showed that there were positive correlations among trait mindfulness, attention, and working memory and that attention had a partial mediating effect between trait mindfulness and working memory, verifying Hypothesis 1. It can be seen that, on the one hand, the higher the level of trait mindfulness, the more concentrated and stable the individual’s attention can be when completing the task, thus better promoting working memory. On the other hand, trait mindfulness can also directly affect working memory levels.

The close relationship between trait mindfulness and attention was consistent with the results from prior studies. It has been found that the human mind wanders for nearly half of its waking time (Killingsworth and Gilbert, 2010), and trait mindfulness and mindfulness training are associated with reduced mind wandering (Mrazek et al., 2012, 2013). Mindfulness controls attention by reducing habitual distribution of attention and attention to distracting information (Wadlinger and Isaacowitz, 2011). It is worth noting that the two mindfulness facets of “observe” and “actaware” were positively correlated with performance on the attention test in study 1, suggesting that these two facets may be the important factors of trait mindfulness affecting attention. The higher the level of trait mindfulness, the more consciously the individual can observe and pay attention to all kinds of experiences, pay more attention to and be aware of their current actions, and then have better performance when completing the attention task test.

In this study, trait mindfulness was found to be related to working memory and positively influenced working memory performance. This is consistent with the results of previous studies (Phillipot and Segal, 2009; Satish et al., 2018). In the process of the influence of trait mindfulness on working memory, 31.60% were realized through the mediation of attention. This is because attention plays a fundamental role in cognitive processes, and this is very important in working memory. It is the first step of information processing by which external information enters the current processing range through attentional selection. On this basis, the further processing of information can be completed by working memory (Cowan et al., 2006). The results of this study also showed that the two mindfulness facets of “observe” and “actaware” are positively correlated with working memory. As mentioned before, these two facets are also positively correlated with attention. In conclusion, these two mindfulness facets can influence working memory through the mediation of attention.

Our results showed that the direct effect of trait mindfulness on working memory was 0.40, accounting for 68.40% of the total effect. This result indicates that apart from attention, trait mindfulness may directly or indirectly affect working memory through other mediating factors and that these effects were greater than those of attention. However, so far, the direct psychological mechanism of mindfulness affecting working memory is still unclear. This study showed that the “describe” facet of trait mindfulness is positively correlated with working memory, but not with attention, suggesting that the “describe” facet may be one of the internal mechanisms by which trait mindfulness directly affects working memory. The higher the “describe” facet score, the more likely the individual is to describe and mark internal experiences with language in daily life (Baer et al., 2006). When taking part in the working memory test, such individuals tend to use internal language to describe and mark the figures in the task. This internal processing strategy helps to make memory clearer and stable, resulting in better working memory test scores. However, it should be noted that the correlation coefficient between the “describe” facet and working memory performance is 0.21, a low-level correlation. Therefore, the direct impact of trait mindfulness on working memory must have other psychological mechanisms, which need further study in the future.

In addition to the above, the reason that trait mindfulness can positively predict attention and working memory may also be related to brain function and brain structure. Most previous research has focused on the impact of mindfulness training on the brain, and only several limited studies evaluating the neural basis of trait mindfulness have shown that trait mindfulness may be related to brain regions involved in attention and working memory, such as the hippocampus (Taren et al., 2013), the double lateral anterior cingulate cortex (ACC) (Lu et al., 2014), etc., and that brain function, neuroelectric activity, and even the structure of these brain areas and other areas related to attention and working memory can be changed through mindfulness practice (Cahn et al., 2010; Wang and Huang, 2011). Therefore, it can be speculated that mindfulness traits may be one of the important psychological factors in understanding the effects of mindfulness exercises on attention and working memory. That is to say, the positive influence of mindfulness exercise on attention and working memory may be achieved by improving the level of trait mindfulness and inducing changes in the corresponding brain structures and functions.

The experimental results of study 2 showed that the attention and working memory scores of the high trait mindfulness group were higher than those of the low trait mindfulness group across all three stress situations (no stress, single stress, and multiple stresses), verifying Hypothesis 4. These results also further deepened the results of study 1. They showed that the positive correlation and influence of trait mindfulness on attention and working memory were consistent across contexts; i.e., whether the stress intensity was great or not, the performance of attention and working memory from those with high trait mindfulness was always better than that from those with low trait mindfulness. These results showed that the level of trait mindfulness can be used to predict the performance of attention and working memory in different stress situations.

In study 2, we also found that stress situations and trait mindfulness interact in the process of influencing attention and working memory. Further simple effect analysis showed that the effects of different stress situations on attention and working memory are regulated by trait mindfulness, verifying Hypothesis 5. To be specific, based on the attention test scores under different stress situations, the relative performance of the high trait mindfulness group on the attention test were as follows: single stress > multiple stresses > no stress. However, for the low trait mindfulness group, although no statistical difference was found in attention under the different stress conditions, it can be seen in Table 3 and Figure 4 that attention had a downward trend in the process from no stress to single stress and multiple stresses. There was no significant change in the test results for working memory across the different stress situations for the high mindfulness group. The working memory performance of the low mindfulness group decreased significantly with the increase of stress intensity.

According to the Yerkes–Dodson law, stress and task performance show an inverted U-shaped curve. When the pressure is moderate, the task performance is the highest. Excessive stress will lead to a decline in task performance. However, whether a stress situation constitutes excessive pressure and how much influence it will have are affected by many factors. According to the interaction theory of stress coping, the influence of stress on individuals is related not only to changes in the stimulating environment or events but also to psychological factors such as individual cognition and coping style (Lazarus and Folkman, 1984). The results of this study showed that trait mindfulness is an important psychological factor that regulates the influence of stress on individual attention and working memory.

For the low trait mindfulness group, working memory decreased significantly with the increase in stress intensity. Although the performance of attention did not show a significant difference, there was a downward trend. This shows that for such individuals, the single stress and multiple stress levels established in this experiment exceed the highest stress level that the participants could bear, resulting in a decline of working memory and attention. Therefore, it is suggested that for such individuals, the degree of stress tolerance is very limited, and a small increase in stress may have a strong negative impact on them. Therefore, in normal learning tasks, time pressure and other factors that could readily cause stress should be reduced, to avoid damaging working memory and attention.

It has been found that increased trait mindfulness can reduce stress sensitivity (Greeson and Gabrielle, 2018). Individuals with high trait mindfulness have less stress sensitivity and better adaptability than those with low trait mindfulness (Grover et al., 2016; Lomas et al., 2017). For high trait mindfulness individuals, the single time stress situation used in this study is close to the appropriate range of stress (the highest point of the inverted U curve). This may enable individuals to reach or approach the best level of arousal, stimulate individual potential, and fully mobilize attention. This helps the individual to complete the attention test quickly and accurately and to make the score on the attention test reach or approach the best possible level. Under the multiple stress condition, the stress intensity exceeds the individual’s ability to bear properly, and so, test scores decline. However, when the individual is in a stress-free situation, the potential of the individual, including attention and other cognitive resources, is not effectively mobilized, yielding the lowest score for all three conditions. Therefore, for the high trait mindfulness group, in the normal learning tasks, increasing the stress intensity appropriately, such as increasing the time pressure properly, will be more conducive to the individual’s concentration and to faster and better completion of the task. In the working memory test, with the increase in stress intensity, the performance of working memory in the high trait mindfulness group was not damaged, indicating that high trait mindfulness had a protective effect on the working memory of individuals coping with stress.

This study yielded another interesting and noteworthy result. Regardless of whether the participant was in the low trait mindfulness group or the high trait mindfulness group, as the stress situation changed, the changes in individual working memory were not synchronized with changes in attention. For example, in the low trait mindfulness group, as the stress increased, working memory decreased significantly, but the performance of attention did not change significantly. In the high trait mindfulness group, the relative scores on the attention test, across all three stress conditions, were as follows: single stress > multiple stresses > no stress, but there were no significant differences in scores for working memory. This may be due to the fact that, although attention is an important factor affecting working memory (Oberauer, 2019), working memory may also be affected by other factors such as emotions (Eysenck et al., 2005) and expectation-driven changes in cortical functional connectivity (Bollinger et al., 2010). Moreover, these factors may have different effects on working memory in different stressful situations, which may lead to the observed asynchrony of the attention and working memory test results.

In conclusion, this study suggests that a good way to improve attention and working memory in different stress situations would be to improve the level of trait mindfulness. Therefore, this study provides a strong theoretical basis and practical guidance for improving attention and working memory.

### Limitations and Future Research

Although this study produced some important findings, it nonetheless has some limitations. First, with regard to research methods, study 1 adopted a cross-sectional design, and thus, it could not draw any conclusions regarding causality; this should be borne in mind when interpreting the results from study 1. Second, with regard to the research group, the subjects selected in this study were all junior school students, from a single sample source. The research conclusions may not be applicable to other groups, raising potential concerns about external validity. Third, with regard to the research content, although this study discussed the possible role of the brain in the process by which trait mindfulness affects attention and working memory, that was only a deduction and no substantive research on that specific topic was conducted.

Therefore, the following aspects need to be further explored in the future. First, a longitudinal study or combined mindfulness interventions should be considered to explore the dynamic relationship among trait mindfulness, attention, and working memory, so as to further verify the causal relationship among them. Second, attention and working memory are very important not only for junior school students but also for other groups. Therefore, one could further expand the scope of the subjects and thereby yield meaningful knowledge for promoting the attention and working memory performance of other groups. Finally, studies on the relationship between brain function and structure and trait mindfulness are still rare, and this aspect should be studied to further clarify the effective mechanisms by which trait mindfulness affects attention and working memory.

## Data Availability Statement

The datasets presented in this article are not readily available because of confidentiality and ethical restrictions. Requests to access the datasets should be directed to the corresponding author.

## Ethics Statement

The studies involving human participants were reviewed and approved by the Harbin Engineering University Ethics Committee. Written informed consent to participate in this study was provided by the participants’ legal guardian/next of kin.

## Author Contributions

YL was responsible for writing the manuscript and analyzing the data. NY was responsible for designing the research and collecting the data. YZ conducted the experiment and reviewed and revised the manuscript. WX took part in designing the research and collecting the data. LC took part in collecting the data. All authors approved the above version to be published.

## Conflict of Interest

The authors declare that the research was conducted in the absence of any commercial or financial relationships that could be construed as a potential conflict of interest.
